# Colorectal cancer patient’s self-efficacy for managing illness-related problems in the first 2 years after diagnosis, results from the ColoREctal Well-being (CREW) study

**DOI:** 10.1007/s11764-017-0636-x

**Published:** 2017-08-19

**Authors:** Chloe Grimmett, Joanne Haviland, Jane Winter, Lynn Calman, Amy Din, Alison Richardson, Peter W. F. Smith, Claire Foster

**Affiliations:** 1 Macmillan Survivorship Research Group, Faculty of Health Sciences, University of Southampton, Southampton, SO17 1BJ UK; 2 The Institute of Cancer Research, 15 Cotswold Road, Sutton, London, SW7 3RP UK; 3 University Hospital Southampton NHS Foundation Trust, Southampton, SO16 6YD UK; 4 Faculty of Health Sciences, University of Southampton, Southampton, SO17 1BJ UK; 5 Social Statistics and Demography, Social Sciences, University of Southampton, Southampton, SO17 1BJ UK

**Keywords:** Colorectal, Cancer, Self-efficacy, Self-management

## Abstract

**Purpose:**

There is a growing emphasis on self-management of cancer aftercare. Little is known about patient’s self-efficacy (confidence) to manage illness-related problems and how this changes over time. This paper describes the patterns of self-efficacy for managing illness-related problems amongst colorectal cancer patients in the 2 years following diagnosis.

**Methods:**

In this prospective cohort study, questionnaires were administered at baseline (pre-surgery), 3, 9, 15 and 24 months to 872 colorectal cancer patients. Self-efficacy (confidence to manage illness-related problems), anxiety, social support, affect, socio-demographics, physical symptoms and clinical and treatment characteristics were assessed. Group-based trajectory analysis identified trajectories of self-efficacy up to 24 months and predictors.

**Results:**

Four trajectories of self-efficacy were identified: group 1 (very confident) 16.0% (95% confidence interval (CI) 10.7–21.3%), group 2 (confident) 45.6% (95% CI 40.3–51.0%), group 3 (moderately confident) 29.5% (95% CI 25.1–33.8%) and group 4 (low confidence) 8.9% (95% CI 6.4–11.4%). Greater deprivation, domestic status, more co-morbidities, worse fatigue and pain, lower positivity and greater negativity were significantly associated with lower self-efficacy. There was an increase in mean scores for self-efficacy over time for the whole sample, but this did not reach the cut-off for minimally important differences. At 2 years, the lowest level of confidence to manage was for symptoms or health problems.

**Conclusion:**

Around 40% of patients had suboptimal levels of confidence to manage illness-related problems with little change from the time of diagnosis across the four groups.

**Implications for cancer survivors:**

Screening for self-efficacy at diagnosis would enable targeted, early intervention which could in turn enhance health-related quality of life.

## Introduction

Cancer is increasingly being viewed as a chronic health condition with evidence that cancer and its treatment can have a significant impact on people’s lives in the months and years following treatment [1–3]. This is reflected in a growing emphasis on self-management of cancer aftercare. Here, the patient is expected to manage the physical and psychosocial consequences of cancer and its treatment, seeking support from the health care system when they feel it necessary, and making lifestyle changes where appropriate to improve health and well-being.

Identifying factors associated with a person’s capability to self-manage after cancer can enhance understanding of how and when to best support them. Self-efficacy, defined by Bandura as ‘beliefs in one’s capabilities to organize and execute the courses of action required to manage prospective situations’ [4], has been identified as a personal resource which may promote adjustment to cancer and other chronic illnesses and is amendable to change.

Data from cross-sectional studies support a positive association between self-efficacy and quality of life [5], adjustment following a cancer diagnosis [6, 7] and symptom distress [8]. Self-efficacy to manage illness-related problems may also moderate the relationship between a physical symptom and quality of life. In a study of 112 women receiving adjuvant endocrine therapy, increasing physical symptoms were only associated with lower functional and emotional well-being in those reporting low self-efficacy for coping with symptoms [9].

There are limited data describing if and how self-efficacy changes over time in cancer populations. Knowledge of patterns of change in self-efficacy from diagnosis and in the years following could highlight the best time to intervene to support those who might be struggling. Existing longitudinal studies tend to focus on breast cancer or mixed cancer populations, with limited data available in colorectal cancer patients. They also have methodological limitations, considerable variability in sample characteristics, and unsurprisingly, reach conflicting conclusions.

Conflicting data exists as to the stability of self-efficacy over time in cancer populations. In one of the earliest studies, Lev and colleagues [10] followed a cohort of 307 cancer patients for 8 months and concluded that mean self-care self-efficacy reduced over time. However, the sample included patients with metastatic disease and less than half completed the 8-month follow-up assessment. In contrast, Manne et al. [5] and Rottman et al. [11] describe cancer-related self-efficacy and general self-efficacy, respectively, remained stable over a 12-month period. However, in a sample of 95 breast cancer patients over a period of 12 months, the exception was the subscales of self-efficacy for activity management and self-satisfaction increased [5]. This study employed the Stanford Inventory of Cancer Patient Adjustment (SICPA) scale. The authors acknowledge that, unlike the other subscales, for the two facets of self-efficacy which improved, respondents were asked to assess confidence in their abilities to manage activities compared to before their diagnosis. This may account for the difference. It should also be noted that the psychometric properties of SICPA have not been confirmed.

Finally, in a more recent study, Mystakidou and colleagues followed 90 cancer patients receiving radiotherapy, assessing their general perceived self-efficacy before radiotherapy commenced and 1 month after completion of their treatment [12]. They recount self-efficacy levels reduced during this time, and were accompanied by an increase in anxiety and deterioration in quality of life. However, the sample size was small, follow-up short and cancer type varied, limiting generalisability.

As well as methodological concerns and sample heterogeneity of the aforementioned studies, they also vary considerably regarding the definition and measure of self-efficacy used. This includes instruments assessing perceived *general* self-efficacy (e.g. the General Self-Efficacy scale [13]) to more specific measures of self-efficacy to cope with cancer-related problems, such as the Cancer Behavior Inventory [14]. We argue, in line with Bandura’s definition, that self-efficacy is a situation-specific competence belief [15], and as others have said [9] that the specificity of the measures used in this context is of utmost importance. Previous data demonstrate that even within a focussed measure of self-efficacy, the Self-Efficacy for Managing Chronic Disease scale [16], there is substantial variation in patient’s perceived ability to manage varying aspects of their illness. For example, self-efficacy to manage fatigue was significantly lower than self-efficacy to access information in a cross-sectional sample of cancer survivors within 1 year of cancer treatment [17]. If the most pertinent targets for intervention are to be determined in the changing landscape of self-management support after cancer treatment, we need to first understand the specific areas of disease self-management that require most support, who is most likely to need support and when.

Previous literature is limited by the reporting of mean self-efficacy scores for the populations under investigation, potentially masking groups of patients with higher or lower self-efficacy. Recently, trajectories of psychosocial and quality of life outcomes have been reported. These analyses determine whether there are distinct groups within the sample, i.e. those scoring consistently low/high during the period of follow-up, which would be masked by group mean scores. In a prospective cohort of colorectal cancer survivors followed from 5 months to 5 years post-diagnosis, Dunn et al. [18] report four trajectories of psychological distress with 19% of the sample reporting consistently low levels of distress. This analytical method was also used by our research group to examine trajectories of quality of life and well-being in the CREW cohort: a large representative group of colorectal cancer patients being treated with curative intent from the point of diagnosis at regular intervals for 2 years. Using the Quality of Life in Adult Cancer Survivors (QLACS) inventory to measure quality of life, we found four distinct trajectories of recovery with two groups faring consistently well (above the median), and two groups (below the median) who consistently struggled [19].

The current study aims to overcome some of the limitations of the previous literature, reporting data from the CREW cohort study. In addition to a prospective longitudinal study design with a pre-surgical baseline assessment, we use a measure of self-efficacy specific to the management of chronic illness: the Self-Efficacy for Managing Chronic Disease scale [16]. The objectives of this paper are to describe patterns of self-efficacy for managing illness-related problems using trajectory analysis, identify areas where self-efficacy is lowest and examine the baseline predictors of self-efficacy in the first 2 years since diagnosis.

## Methods

### Design

CREW is a prospective, longitudinal cohort study with patients recruited from 29 UK hospitals prior to surgery with curative intent colorectal cancer. Full details of the CREW study protocol can be found elsewhere [20].

### Participants

Eligibility criteria included (a) a diagnosis of colorectal cancer (Dukes A–C), (b) awaiting primary surgery with curative intent, (c) ≥ 18 years old and (d) had the ability to complete questionnaires (language line translation facilities were available for those that did not speak English). Exclusions included distant metastatic disease at diagnoses or a prior diagnosis of cancer (other than non-melanomatous skin cancer or in situ carcinoma cervix).

### Procedure

Written informed consent was obtained and the UK National Health Service National Research Ethics Service (REC reference number 10/H0605/31) approved conduct of the study. Eligible patients were identified by a member of their clinical care team during multidisciplinary team meetings at one of the 29 UK cancer centres involved in CREW. Baseline questionnaires were completed prior to primary surgery wherever possible with subsequent follow-up at 3, 9, 15 and 24 months post-surgery and annual assessments are ongoing. Clinical and treatment details were gathered (with consent) from medical notes. This included stage, type and grade of disease as well as details of type of surgery, chemotherapy and radiotherapy. Postcode was used to derive the Index of Multiple Deprivation, representing neighbourhood deprivation.

### Measures

Areas of assessment were informed by our recovery framework describing constructs believed to be important in recovery from cancer diagnosis (see [21] for full details). Validated measures were repeated at every time point unless otherwise indicated.

*Self-efficacy* was assessed at all time points using the Self-efficacy for Managing Chronic Disease (SEMCD) scale [16]. From baseline to 9 months inclusive, the original six-item version of the scale was used; from 15 months onwards, an additional five cancer-specific items were included, comprising the Cancer Survivors’ Self-Efficacy Scale (CS-SES [22]). The CE-SES was found to have excellent reliability (Cronbach’s alpha 0.92) with the 11-item scale) [22]. At 2 years, the term ‘your disease’ was changed to ‘your cancer’ throughout this measure to ensure respondents were relating the items to their cancer experience rather than other co-morbid conditions. For example, the item ‘How confident are you that you can keep the emotional distress caused by *your disease* from interfering with the things you want to do?’ was changed to ‘How confident are you that you can keep the emotional distress caused by *your cancer* from interfering with the things you want to do?’ All items are measured on a scale from 1 (not at all confident) to 10 (totally confident), and an overall score is obtained by taking the mean of all the individual items.

*State anxiety* was measured using the State-Trait Anxiety Inventory (STAI [23]) consisting of 20 items. Higher scores indicate greater anxiety. Scores ≥ 40 suggest clinically significant anxiety.

*Depression* was assessed using the 20-item Centre for Epidemiological Studies Depression (CES-D [24]) scale. Higher scores indicate greater depression and scores of ≥ 20 suggest clinical depression [25].

The Medical Outcomes Study (MOS) measured *social support*. The scale includes 19 items, 18 of which comprise four subscales: emotional/informational, tangible, affectionate support and positive social interaction. Higher scores denote greater social support [26].

Symptoms of *pain and fatigue* were examined using the subdomains of the QLACS scale, each subdomain consisting of four items. Higher scores indicate worse pain and fatigue [27]. These symptoms were highly prevalent and two of the items of the SEMCD scale refer specifically to pain and fatigue.

*Positive and negative affect* was assessed by the Positive and Negative Affect Schedule (PANAS) Short Form [28]. Two five-item mood scales measure positive and negative affects with higher scores depicting stronger positive or negative emotions.

### Statistical methods

Published guidance for dealing with missing items in subscales were applied where available; otherwise, providing at least 75% of individual items within a subscale had been completed. The scores of any missing items within the scale were taken to be the mean of the scores of the available items. Participants with missing questionnaires, including baseline, were included in analyses for time points for which they provided data, and there was no imputation of missing questionnaires from other completed questionnaires. The Index of Multiple Deprivation was categorised into quintiles.

Cross-sectional analyses summarised mean scores for the overall self-efficacy scales (6- and 11-item versions), as well as individual items from baseline to 2 years following surgery. Changes in the overall mean score and individual items of the six-item scale from baseline to 2-year follow-up of at least half a standard deviation (SD) were assessed as minimally important differences [29].

Group-based trajectory analyses [30] were used to investigate whether distinct groups could be identified for the six-item SEMCD scale from baseline to 24 months. These are discrete mixture models, which modelled the SEMCD score as censored normal data following a polynomial time curve. The optimal number of distinct trajectories was determined using the Bayesian information criterion (BIC) [31, 32] to compare model fit (a change in BIC > 10 supports the more complex model), whilst aiming to avoid trajectories containing very few individuals. The shape of each trajectory was assessed to determine whether it was best described by a linear, quadratic or cubic function according to the significance of each term. Estimated proportions of participants within each trajectory were obtained from the models, with 95% CIs. Potential predictors of trajectory group membership were included in the models within the following domains: (a) socio-demographic factors at baseline (age, gender, domestic status, neighbourhood deprivation), (b) clinical and treatment factors (tumour site, Dukes stage, co-morbidities, neoadjuvant and adjuvant treatment, stoma), (c) physical symptoms at baseline (fatigue and pain) and (d) psychosocial factors at baseline (positive and negative affects, anxiety, depression, social support). All factors found to be statistically significant (*p* < 0.05) for at least one of the trajectories were then included together in an overall model to find which factors remained independent significant predictors of group membership. Statistical significance of model parameters was assessed by the Wald test. For predictors of trajectory group membership, the following were included as continuous variables in the model: age, fatigue, pain, positive and negative affects, overall social support and the categorical data fitted as factors: gender, domestic status, neighbourhood deprivation, clinical and treatment factors, anxiety and depression.

Date of surgery was taken as time zero and follow-up time calculated using date of questionnaire completion; timing of baseline questionnaire (pre-/post-surgery) was adjusted for in all trajectory models as approximately 30% of participants completed their baseline after surgery due to logistical reasons, including emergency surgery. To ease interpretation of the estimated trajectories, the following cut-offs were assigned to the six-item SEMCD scale: 1–4 ‘low confidence’, 5–6 ‘moderate confidence’, 7–8 ‘confident’ and 9–10 ‘very confident’.

## Results

### Study participants

A total of 857 participants consented to follow-up (excluding 15 who withdrew at baseline). Response rates of those remaining eligible for questionnaires at each time point were 88% at baseline, 84% at 3 months, 82% at 9 months, 80% at 15 months and 74% at 24 months at the point of analysis. Full details of participants are described elsewhere [19]. In brief, the sample comprised 65% colon and 35% rectal cancer patients; disease stage was 14% Duke’s A, 53% Duke’s B and 32% Duke’s C; 18% received neoadjuvant and 35% adjuvant therapy; and 35% had a stoma (most temporary). The mean age of participants was 68 years, with 60% male. By 2 years, 79 (9.9%) had experienced a recurrence, 65 (7.6%) had died and 105 (12.2%) had withdrawn.

### Levels of self-efficacy over follow-up

Self-efficacy scores (overall mean scores and for individual items of 6-item SEMCD scale and 11-item CS-SES scale) are shown in Table 1. For the group as a whole, there was evidence of an increase in self-efficacy over the 2-year follow-up, with statistically significant changes in the overall mean score (*p* < 0.001) and some individual scores (*p* < 0.01 for all subscores on the six-item scale), although the absolute change in scores was small. The mean score of the six-item SEMCD scale increased from 7.4 (SD = 1.9) at baseline to 8.2 (SD = 1.8) at 2 years, not quite reaching the cut-off for a minimally important difference (MID; 0.5 × SD).

**Table 1:** Descriptive results for self-efficacy from baseline to 2 years following surgery

Mean (SD) shown; range is 1–10 for all scales and individual items	Baseline (*N* = 756)	3 months (*N* = 668)	9 months (*N* = 623)	15 months (*N* = 579)	24 months (*N* = 514)
6-item SEMCD scale	7.4 (1.9)	7.6 (2.0)	7.8 (1.9)	8.0 (1.8)	8.2 (1.8)
11-item CS-SES scale	N/A	N/A	N/A	8.2 (1.7)	8.3 (1.6)
Self-efficacy (confidence) to manage					
Fatigue	7.2 (2.2)	7.3 (2.3)	7.5 (2.2)	7.9	8.1 (2.1)
Physical discomfort or pain	7.2 (2.1)	7.6 (2.2)	7.7 (2.2)	8.1 (2.0)	8.4 (1.9)
Emotional distress	7.3 (2.2)	7.7 (2.2)	7.9 (2.1)	8.2 (2.0)	8.4 (1.9)
Symptoms or health problems	7.4 (2.2)	7.4 (2.2)	7.5 (2.2)	7.8 (2.1)	7.7 (2.2)
Different tasks and activities	7.7 (2.0)	7.9 (2.0)	8.0 (2.0)	8.2 (1.9)	8.2 (2.0)
Do things other than just take medication	7.8 (2.1)	7.8 (2.1)	8.0 (2.1)	8.1 (2.0)	8.2 (2.0)
Access information	N/A	N/A	N/A	8.3 (2.0)	8.5 (1.9)
Access people to help and support	N/A	N/A	N/A	8.4 (2.0)	8.5 (1.9)
Deal with problems by yourself	N/A	N/A	N/A	8.0 (2.1)	8.1 (2.0)
Contact doctor	N/A	N/A	N/A	8.5 (2.0)	8.6 (2.0)
Get support with problems	N/A	N/A	N/A	8.2 (2.1)	8.3 (2.1)

*N* indicates the number of questionnaires returned at each time point; the completeness of data within questionnaires varies; e.g. the overall mean score for the six-item SEMCD/CS-SES scales was available for 741 participants at baseline, 656 at 3 months, 610 at 9 months, 568 at 15 months and 505 at 24 months. The extent of missing data on individual items ranged from 0.8% for the item ‘how confident are you that you can keep the physical discomfort or pain of having had cancer and/or cancer treatment from interfering with the things you want to do?’ at 24 months to 8.1% for the item ‘How confident are you that you can deal by yourself with the problems cancer and/or cancer treatment has caused?’ at 15 months

*SD* standard deviation, *SEMCD* Self-efficacy in Managing Chronic Disease, *6 items* original Lorig scale (measured at all time points), *11 items* original six-item Lorig scale plus five additional cancer-specific items (measured at 15 and 24 months), *N/A* not available

At baseline, participants reported lowest levels of confidence with fatigue and physical discomfort/pain, and highest level for ‘doing things other than just taking medication’. These improved over the 2 years in the group as a whole. At 2 years, the lowest level of confidence to manage was for symptoms or health problems and the highest for contacting the doctor. Comparing scores between baseline and 24 months for individual items, the following reached the above definition of a MID: physical discomfort/pain and emotional distress, with a borderline change for fatigue (Table 1).

**Fig. 1:**
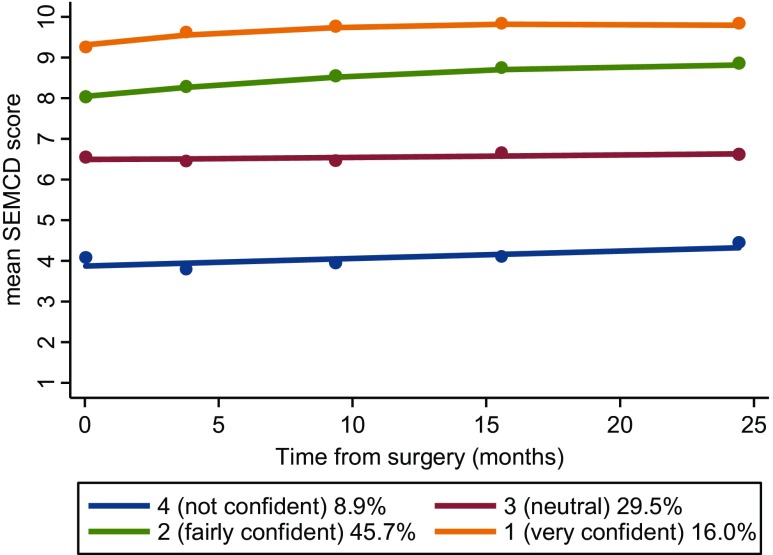
Estimated trajectories of mean six-item SEMCD score from baseline to 24 months after surgery for 802 CREW participants with self-efficacy data

The optimal number of distinct trajectories (groups) identified for the six-item SEMCD scale from surgery to 2 years was 4 (change in BIC from three groups to 4 = 165.57 and change in BIC from four groups to 5 = 9.73). Estimated proportions of the CREW sample in each group were 16.0% (95% CI 10.7–21.3%) in group 1 (very confident, with highest levels of self-efficacy in CREW), 45.6% (95% CI 40.3–51.0%) in group 2 (confident), 29.5% (95% CI 25.1–33.8%) in group 3 (moderate confidence) and 8.9% (95% CI 6.4–11.4%) in group 4 (low confidence, with lowest levels of self-efficacy) (Fig. 1). The small increase in levels of self-efficacy over follow-up found in the whole group was less apparent when examining the trajectories (Fig. 1), suggesting no clinically significant improvements over the 2-year follow-up.

### Associations with self-efficacy

From the trajectory models including potential predictors of self-efficacy, the following baseline factors were found to be significantly associated with lower levels of self-efficacy over the 2-year follow-up: greater neighbourhood deprivation, domestic status (being single/widowed/divorced/separated), a greater number of co-morbidities, worse symptoms of fatigue and pain, lower positivity and greater negativity (Table 2). Those who had a stoma also reported lower levels of self-efficacy. These characteristics showed statistically significant differences for at least one of the lower self-efficacy groups (groups 2, 3, 4) compared with the reference trajectory (group 1 = very confident) in the multiple regression model. For the comparison of group 4 (lowest self-efficacy) versus group 1 (highest self-efficacy), all of the listed participant characteristics were highly statistically significant (*p* < 0.01) except for neighbourhood deprivation (Table 2). There were no significant associations with self-efficacy group for age, gender, tumour site, Dukes stage, neoadjuvant and adjuvant treatment, anxiety, depression and social support.

**Table 2:** Associations between baseline participant characteristics and level of self-efficacy (confidence to self-manage aspects of illness) up to 2 years after surgery: results from final trajectory model

Domain	Baseline characteristic	Trajectory of six-item SEMCD scale			
		Group 1: very confident (*N* = 116)	Group 2: fairly confident (*N* = 388)	Group 3: neutral (*N* = 232)	Group 4: not confident (*N* = 66)
Socio-demographic	Neighbourhood deprivation quintile	Reference	*p* = 0.06	*p* = 0.03	*p* = 0.13
	First (least deprived)	28 (24.8%)	84 (22.2%)	39 (17.0%)	9 (14.1%)
	Second	26 (23.0%)	89 (23.5%)	41 (17.8%)	9 (14.1%)
	Third	20 (17.7%)	71 (18.8%)	47 (20.4%)	12 (18.7%)
	Fourth	23 (20.3%)	61 (16.1%)	50 (21.7%)	14 (21.9%)
	Fifth (most deprived)	16 (14.2%)	73 (19.3%)	53 (23.0%)	20 (31.2%)
	Domestic status	Reference	*p* = 0.16	*p* = 0.01	*p* = 0.002
	Married/living with partner	87 (82.1%)	273 (74.8%)	138 (63.9%)	31 (51.7%)
	Single/widowed/divorced/separated	19 (17.9%)	92 (25.2%)	78 (36.1%)	29 (48.3%)
Clinical and treatment	Co-morbidities	Reference	*p* = 0.60	*p* = 0.22	*p* < 0.001
	None	42 (40.0%)	107 (34.2%)	23 (12.4%)	10 (19.2%)
	1	45 (42.9%)	88 (28.1%)	61 (32.8%)	16 (30.8%)
	2	13 (12.4%)	68 (21.7%)	61 (32.8%)	7 (13.5%)
	3+	5 (4.8%)	50 (16.0%)	41 (22.0%)	19 (36.5%)
	Stoma	Reference	*p* = 0.01	*p* < 0.001	*p* < 0.001
	No	88 (76.5%)	255 (66.7%)	130 (57.3%)	31 (47.7%)
	Yes	27 (23.5%)	127 (33.3%)	97 (42.7%)	34 (52.3%)
Symptoms	Fatigue; mean (SD)	Reference	*p* < 0.001	*p* < 0.001	*p* < 0.001
		9.3 (5.0)	11.9 (5.0)	14.8 (4.8)	18.9 (4.7)
	Pain; mean (SD)	Reference	*p* = 0.13	*p* < 0.001	*p* < 0.001
		6.7 (3.4)	8.9 (4.7)	11.4 (5.7)	14.6 (6.5)
Psychosocial	Positivity; mean (SD)	Reference	*p* = 0.05	*p* < 0.001	*p* < 0.001
		20.2 (5.3)	19.1 (4.0)	16.1 (3.9)	15.0 (3.5)
	Negativity; mean (SD)	Reference	*p* = 0.02	*p* < 0.001	*p* < 0.001
		6.7 (2.6)	7.8 (3.1)	10.1 (3.6)	12.2 (4.8)

Final trajectory model included all participant characteristics shown in table (i.e. those statistically significant for at least one group comparison) as well as a term indicating whether baseline questionnaire completed before or after surgery. *Higher* scores for fatigue and pain indicate *greater* problems, range 4–28 (QLACS subscales). *Lower* scores for positivity and greater scores for negativity indicate *poorer* levels of personal affect, range 5–25 (PANAS subscales). *P* values represent significance of Wald test for each characteristic in groups 2, 3 and 4, respectively, compared with group 1 (reference group). Denominators for percents vary due to missing data for some characteristics; percents are calculated out of those with data available. Extent of missing data for predictor variables: fatigue *N* available 753, *N* missing 3; pain *N* available 748, *N* missing 8; positivity *N* available 724, *N* missing 32; negativity *N* available 731, *N* missing 25 at baseline. Cut-offs used to describe categories of mean six-item SEMCD scale: 1–4 ‘low confidence’, 5–6 ‘moderately confident’, 7–8 ‘confident’ and 9–10 ‘very confident’

*SD* standard deviation

## Discussion

This paper reveals for the first time that across group trajectories, levels of self-efficacy remain stable in the first 2 years following colorectal cancer surgery with curative intent (pre-surgery and 3, 9, 12 and 24 months later). Sixty-two percent of the participants reported feeling very/fairly confident to manage their illness-related problems in the first 2 years. A third of the population reported feeling neither confident nor lacking in confidence, and the remaining 9% lacked confidence to manage illness-related problems over the 2 years. This suggests that around 40% of those treated with curative intent for colorectal cancer could benefit from the offer of additional self-management support to manage illness-related problems.

Exploring areas where people feel least confident to manage is important to guide specific areas for intervention. When looking at the CREW population as a whole, our results demonstrate clinically meaningful improvements in mean scores across two of the six individual items, namely, confidence to manage physical discomfort/pain and emotional distress. In contrast, self-efficacy for managing other symptoms and health-related problems showed very little change and was the area of lowest reported confidence at 2 years.

There are few comparable published studies describing domain specific confidence to manage disease-related problems. In a cross-sectional sample of 182 cancer patients within 1 year of treatment, Foster et al. [17] report similar findings with lowest levels of confidence for fatigue, emotional distress and symptoms in the first 12 months post curative intent treatment across a range of cancer types.

Due to variations in how self-efficacy is defined and measured across studies, and the discordance in populations and timing of assessments, there is little agreement in previous studies with regard to the pattern of self-efficacy over time with some authors reporting a decline [10, 12, 33] and others recounting little change over a 12-month period [5, 11]. However, none looked specifically at colorectal cancer patients, followed patients for more than 12 months nor used a comparable, psychometrically robust measure of self-efficacy, measuring confidence to manage disease-related problems.

Examination of factors associated with lower levels of self-efficacy revealed those with baseline scores indicating greater deprivation, who were single, had more co-morbidities, worse baseline fatigue or pain and had a stoma as part of their treatment (either reversed or permanent) were more likely to report lower scores. Existing literature has demonstrated an association between depression and anxiety and self-efficacy [34 12]. Whilst we found a univariate association (data not shown), these factors were not predictive of self-efficacy once other variables in the model were taken into account. Also noteworthy is the absence of influence of age or any other disease or treatment variables including site and stage of disease, and whether or not a person had received chemotherapy. These findings are similar to those reported by our group in a cross-sectional sample of cancer survivors [17]. Comparable findings have also been described in a sample of breast cancer patients undergoing adjuvant endocrine therapy. Here, Shelby et al. [9] report that cancer stage, time since surgery and receipt of chemotherapy were not significantly associated with level of self-efficacy as measured by a modified version of the ‘standard efficacy scale’. This challenges the approach of stratified follow-up in cancer care which is entirely determined by disease and treatment-specific factors, with little consideration of psychosocial and symptom-related factors.

There are numerous studies describing the positive cross-sectional association between self-efficacy and quality of life, adaptation to cancer diagnosis and reduced distress in cancer patients [35–38]. Strengthening the argument of the importance of self-efficacy in recovery from cancer, we reported that self-efficacy to manage disease-related problems measured at the time of diagnosis was a significant predictor of quality of life and well-being over the following 2 years in the CREW cohort [19]. Based on this evidence, we suggested that interventions to bolster self-efficacy should be offered as early as possible in order to enhance recovery. Data presented here reinforces this recommendation, as there is little evidence of spontaneous increases in self-efficacy over time. Our data indicate that problems associated with symptoms including pain, fatigue and distress are associated with the lowest self-efficacy scores and suggest important areas for intervention. The question that now needs to be addressed is how might we intervene to support those with least confidence?

Self-management support is a key component in the shift towards stratified aftercare for cancer patients in the UK and has been acknowledged internationally as an integral element to optimise health after cancer [39]. Central to self-management programmes is that patients need to be equipped with the skills, knowledge and, crucially, confidence to enable effective management of the consequences of cancer and its treatment.

There has been increasing research interest in the development and testing of self-management interventions in the cancer domain with a recent systematic review identifying 42 randomised controlled trials examining self-management education [40]. A narrative qualitative synthesis revealed improvements in fatigue, pain, depression, anxiety, emotional distress and quality of life with 90% of identified studies including intervention components that facilitated self-efficacy to manage the consequences of cancer (though few measured this as an outcome). Because there was considerable heterogeneity in the intervention components, a meta-synthesis was not possible, nor agreement on the components of self-management interventions associated with the greatest improvement in outcomes.

Evidence from the aforementioned review does suggest that tailoring self-management interventions to specific problems, for example, depression or distress may lead to favourable outcomes. This is in accordance with our data suggesting the importance of taking account of levels of self-efficacy for specific symptoms. This is echoed in a recently published large randomised controlled trial (RCT) evaluating a nurse-led supportive care package for colorectal cancer survivors [41]. Despite a comprehensive intervention including information, a nurse-led end of treatment session, tailored survivor care plan and telephone follow-up, the study failed to show a significance difference in their primary (distress) or secondary (survivorship care needs and quality of life) outcomes. The authors propose that this was due to the absence of tailoring of the intervention to those most in need, which they suggest should be determined by level of symptom burden. We would argue that symptom burden might not tell the whole story, and that confidence to manage, whatever the level of symptoms, may be just as important.

### Limitations

Ninety-one percent of eligible patients were approached to take part in the study and response rates were high. However, some attrition is evident as would be expected in a longitudinal study with 74% of participants who were still eligible for follow-up returning questionnaire at 24 months. Those patients who declined participation in the CREW study were broadly similar to those who returned questionnaires, although there were fewer older and frail participants consented to the cohort. Those with lower baseline scores of self-efficacy were also less likely to return questionnaires at subsequent time points; thus, our results may underrepresent those with the lowest self-efficacy levels.

## Conclusion

Our analysis has revealed four distinct groups of patients with different levels of self-efficacy. Particular attention is needed for self-management support of symptom-related problems and distress, where confidence to manage was the lowest. Clinicians can also be informed by the factors found to be associated with lower levels of self-efficacy including multi-co-morbidities, having a stoma and living alone, which may help them identify those patients most in need of support. Support for those reporting low confidence should be available close to the time of diagnosis, rather than waiting until the end of treatment. This would require a proactive, rather than a reactive approach to patient care. Almost 40% of participants reported suboptimal confidence to self-manage illness-related problems. Early screening and appropriate intervention could significantly increase self-efficacy and consequently enhance the quality of life and recovery experiences of this substantial group of people living with and beyond colorectal cancer. Further research is required to develop self-management interventions that consider self-efficacy to manage illness-related problems at diagnosis—including symptoms associated with cancer and its treatment as well as co-morbidities. They must be endorsed and supported by health care professionals and implementable within the appropriate health care system.
